# The Lay Press and "Miracles"

**Published:** 1903-05-30

**Authors:** 


### The Lay Press and "Miracles."

Not the least of the difficulties against which practitioners of medicine or surgery have to contend, in their endeavours to obtain correct appreciation of their work from the world in general, is furnished by the surpassing (or as they would themselves call it, the "phenomenal") ignorance of a very large proportion of the well-meaning persons who set up to be public instructors. Of this a very recent instance has been furnished by the case of a poor man living at Bridge of Weir in Renfrewshire, who has been cured of congenital cataract at the age of 30; a case in which the only thing in the least remarkable was that he had not been cured before. Here, we are gravely told by the *Daily Mail*, to select only one example out of many, is a "miracle of science," a phrase which, we venture humbly to submit, is in itself a contradiction in terms. "Science," whatever else it may be, is distinctly human; and a "miracle," according to Dr. Johnson, is "something beyond human power." Our contemporary, possibly in order to justify its own account of the event as given in an "interview" with the patient, attributes to him an acuteness and accuracy of vision, immediately after the operation, that is to say before he was supplied with glasses to supply the place of the natural lenses, and before he could have learned to recognise the nature of the things seen, which would only have been possible, optically speaking, if he had been very highly myopic as well as the subject of cataract, and this, physiologically speaking, unless miraculous, would not have been possible at all. In one respect the case as described by the *Daily Mail* differs, much to the advantage of the patient, from the well-known one which was described, in a still more graphic manner, by the late Thomas Hood. In the latter, as our readers will remember, the man whose sight was restored looked upon his wife, and "saw her very plain"; but, to the newly-restored vision of Mr. Carruth, it is said that all women are beautiful.

If there were any "miracle" at all in the story of which the *Daily Mail* has made so much, we should look upon it as resting upon the fact that at Bridge of Weir, in populous and educated Renfrewshire, some ten miles from Paisley and fifteen or thereabouts from Glasgow, in the latter part of the nineteenth century, it was possible for a child suffering from curable blindness to grow up to manhood without relief; more especially as we are told that there were two other blind children in the family. Was there no "meenister" in the locality? no doctor? no philanthropist? nobody possessed even of a grain of common sense?

When stripped of the meretricious adornments provided by the writers of "pars," these cases of neglected cataract are always curious and interesting, and Dr. Maitland Ramsay is heartily to be congratulated on the good result which he has obtained. It is perhaps to be regretted that the patient was permitted to return to Bridge of Weir so soon, because the gradual education of eyes which have long been sightless offers to the physiologist many valuable opportunities of investigation—opportunities which would, of course, be greater in the case of an adult of average intelligence, than they are in the young children who are the ordinary subjects of operation. An instance has lately fallen under our observation in which the operation was performed, and perfect sight for the first time obtained, at the age of 21; but in this the lenses had never been sufficiently opaque to produce what would be properly called blindness. They were deeply foggy; and, although the patient could neither read nor recognise colours, nor see distant objects, she could always see the outlines of large things near at hand. Such an amount of vision as this has probably existed in nearly all the cases in which cataract has been neglected until adult age was reached, and in such conditions, of course, the improvement of sight obtained, however great, could not be described as "restoration," and would not afford opportunities for studying the dawn of what was practically a new function. We have, however, seen more than one example in young children submitted to operation, of the gradual education of the eyes by the sense of touch. On one such occasion, the child was an inmate of a workhouse, and only eight years old. At a very early stage of the case she was introduced into the board-room, and one of the guardians, a type of the intelligence to which newspaper "pars" minister, suddenly thrust before her a prodigious "gamp" and said "Wot's that?" "Oh, if you please, Sir," said the nurse in charge, who was not wanting in shrewdness, "the little girl has never seen an umbrella, but she has seen money. If you or the other gentlemen would put some pennies on the table, she would see to pick them up fast enough." We need hardly say that the little one went back to her ward with a harvest.

